# Comparison of Weight-for-Height Z-score and Mid-Upper Arm Circumference to Diagnose Moderate and Severe Acute Malnutrition in children aged 6-59 months

**DOI:** 10.12669/pjms.35.2.45

**Published:** 2019

**Authors:** Attia Bari, Mubeen Nazar, Aisha Iftikhar, Sana Mehreen

**Affiliations:** 1 *Attia Bari, MBBS, DCH, MCPS, FCPS (Pediatrics), MHPE Department of Paediatric Medicine, The Children’s Hospital & The Institute of Child Health (CHICH), Lahore, Pakistan*; 2 *Mubeen Nazar, FCPS. (Paediatric Medicine). Department of Paediatric Medicine, The Children’s Hospital & The Institute of Child Health (CHICH), Lahore, Pakistan*; 3 *Aisha Iftikhar, FCPS. (Paediatric Medicine). Department of Paediatric Medicine, The Children’s Hospital & The Institute of Child Health (CHICH), Lahore, Pakistan*; 4 *Sana Mehreen, FCPS. (Paediatric Medicine). Department of Paediatric Medicine, The Children’s Hospital & The Institute of Child Health (CHICH), Lahore, Pakistan*

**Keywords:** Malnutrition, Mid upper arm circumference, Under five children, WHZ score

## Abstract

**Objective:**

To compare weight for height (WHZ) and mid upper arm circumference (MUAC) to diagnose malnutrition in children aged 6–59 months and to determine the association of various risk factors with the nutritional status of the children.

**Methods:**

Descriptive study conducted at the Department of Paediatric Medicine of The Children’s Hospital Lahore from May 2017 to April 2018. A total of 257 children 6 to 59 months of age having MUAC of <125mm were included. WHZ scoring was done and compared with MUAC.

**Results:**

There was slight male predominance 135 (52.5%). Mean age of children was 13.43 + 8.81 months (95% CI: 12.34-14.51). Mean MUAC was 103±13.5 mm (95%CI: 101-105mm). Exclusive breast feeding was present in 82 (32%). Maternal illiteracy was common in SAM (p = was 0.001). More children (73.2%) were identified as SAM by MUAC of <115 mm as compared to WHZ of <-3SD (70%). The ROC curve analysis for MUAC (cut-off value:103, 95%CI; AUC: 101-107 mm) showed it as an excellent predictor (p=<0.001) for children having SAM and WHZ <-3SD, with (AUC= 0.786 [95%CI; 0.725-0.848]).

**Conclusion:**

Both MUAC and WHZ showed fair degree of agreement to diagnose moderate and severe acute malnutrition among children aged 6–59 months. At the community level of resource limited countries, MUAC can be used as an appropriate rapid diagnostic method to identify malnourished children to be managed in nutritional rehabilitation programs.

## INTRODUCTION

Worldwide acute malnutrition is a major public health concern and severely undernourished children are at high risk of mortality. Almost 16 million under 5 children are affected by severe acute malnutrition (SAM) and over half a million die annually. These >500,000 annual childhood deaths can be prevented by in-time management of acute malnutrition in young children.^1^ SAM is caused by either inadequate intake or improper absorption of food. Due to their weakened immune system malnourished children are more susceptible to illness and are nine times more likely to die than well-nourished children.^2^

Children with moderate acute malnutrition (MAM) if not identified timely, can progress into SAM. The main aim of screening program for detecting malnourished children is to prevent mortality. To identify children with malnutrition screening at community level is necessary. Two anthropometric diagnostic methods for diagnosing and referring children with malnutrition for treatment and rehabilitation are MUAC and WHZ.^3^ To identify MAM and SAM, MUAC of 115-125mm and <115 mm respectively is used for community screening.^4^ However, currently it is unclear how MUAC and WHZ are related to each other. Literature review showed that WHZ and MUAC do not always identify the same population of children as having SAM.^3^,^5^

It is imperative for the diagnostic tools to identify correctly children who are at high risk of death due to undernutrition. Due to the conflicting results of WHZ and MUAC in identifying children as SAM, it would be necessary to know how this discrepancy between these two anthropometric measurements is related to identifying children for treatment of SAM in nutritional rehabilitation program. Although multiple studies are available from all over the world but limited research work is done in Pakistan^6^ on this topic so there is a need and room to document our experience about identification of children with MAM and SAM using these two-different diagnostic measures. Thus, we planned this study to describe the distribution of children diagnosed as SAM using (MUAC < 115mm; WHZ <-3SD) and MAM (MUAC 115mm-125mm; WHZ <-2SD), and to assess the degree of agreement between these two diagnostic tools in children aged 6 -59 months at a Tertiary Care Hospital Lahore.

## METHODS

This was a hospital based descriptive study, conducted at The Children’s Hospital Lahore over a period of 1 year from May 2017 to April 2018 after the approval from Institutional Review Board and informed written consent from parents. Sample size was calculated to be 237 by taking 19% under five children as moderately or severely underweight. We had 257 children over one year so we extended our sample. Children 6 to 59 months having MUAC of <125 mm, admitted in medical ward for nutritional rehabilitation were included. A non-stretch tape measure (provided by WHO) was used for measurement of MUAC (to the nearest 1 mm). Weight was measured by a digital scale with the child wearing only light clothes or no clothes and recorded (to the nearest (0.1 kg). Length/height was measured with an “infantometer” (to the nearest 0.1 cm). Anthropometric measurements were then transformed to Z-scores. Malnutrition was categorized into SAM (WHZ score; <-3SD, MUAC; <115mm) and MAM (WHZ score; <-2SD, MUAC; 115mm-125mm). Age of children was confirmed (nearest month) by asking age and date of birth. To minimize data collection errors, data was collected by our qualified and experienced staff nurse attached to the nutrition and rehabilitation ward.

Information about demographic profile, gender, feeding practices and initiation of complementary feeding were noted. Mother’s education status was determined and number of under five children were recorded. Continuous and categorical variables were expressed as mean ± (standard deviation) and number (percentage) respectively. Comparison of proportions were performed with a chi-square test. For comparison of continuous variables, Student’s t-test was used and a p-value of less than 0.05 was considered significant. A receiver operating characteristics (ROC) curve analysis was used to select best cut off value of MUAC in the prediction of SAM. Area under the curve (AUC) and its standard error was calculated. The data was analyzed by using statistical software SPSS-20.

## RESULTS

The results of our study showed predominance of males 135 (52.5%) over females 122 (47.5%). Mean age of children was 13.43±8.81 months (95% CI: 12.34-14.51). Major proportion of patients fell in the age range of 6-11 months constituting 130 (50.5%). The mean age of children having SAM based on MUAC of <115 mm was lower 12.44±8.53, (95% CI; 11.21-13.67) as compared to children with mean age of 13.47±9.60, (95% CI: 12.06-14.88) based on WHZ score of <-3SD. Mean MUAC was 10.33±1.35 cm (95%CI: 10.16-10.50). Exclusive breast feeding was present in only 82 (32%) of children. The main characteristics of the children and their mothers are shown in Table-I.

**Table-I:** Demographics of study participants.

Category	Number (%)
***Age***	
Mean age in months	13.43±8.81
6 months - 11 months	130 (50.5)
12 months - 23 months	96 (37.4)
24 months - 59 months	31 (12.1)
***Sex***	
Male	135 (52.5)
Female	122 (47.5)
***Maternal education***	
Illiterate	137 (53.3)
Primary	83 (32.3)
Secondary	33 (12.8)
Graduate	04 (1.6)
***Type of feeding***	
Exclusive breast feeding	82 (32)
Breast milk + top feeding	95 (37)
Only Cows or formula milk	80 (31)
***MUAC***	
Mean MUAC in cm	10.33±1.35 cm
<11.5 cm	188 (73.2)
115-125cm	69 (26.8)
***WHZ- scoring***	
-2SD	77 (30)
-3SD	180 (70)

The two main factors related to children with SAM having MUAC <115mm were age and maternal educational status (p = <0.001 and 0.001) respectively. Majority 108 (78.8%) mother of children who had MUAC <115mm were illiterate (p = 0.011). The type of feeding had no significant impact on SAM (p = 0.723) (Table-II).

**Table-II:** Association of various factors with nutritional status of under 5 children.

Variables	WHZ score		P-value	MUAC		P-value
	
	<-2SD	<-3SD		<115mm	115-125mm	
***Age***						
6 m-<1 year	28 (21.5%)	102 (78.5%)		111 (85.4%)	19 (14.6%)	
1-<2 years	42 (43.8%)	54 (56.2%)	0.001	58 (60.4%)	38 (39.6%)	<0.001
2-5 years	07 (22.6%)	24 (77.4%)		19 (61.3%)	12 (38.7%)	
***Maternal education***						
Illiterate	35 (25.5%)	102 (74.5%)		108 (78.8%)	29 (21.2%)	
Primary	23 (27.7%)	60 (72.3%)	0.012	61 (73.5%)	22 (26.5%)	0.011
Secondary	18 (54.5%)	15 (45.5%)		17 (51.5%)	16 (48.5%)	
Graduate	01 (25.6%)	03 (75%)				
***Type of feeding***						
Exclusive breast milk	21 (25.6%)	61 (74.4%)		58 (70.7%)	24 (29.3%)	
Breast milk+Top feed	29 (30.5%)	66 (69.5%)	0.522	69 (72.6%)	26 (27.4%)	0.723
Only Top feeding (cows/Formula)	27 (33.8%)	53 (66.2%)		61 (76.2%)	19 (23.8%)	

**Fig.1:**
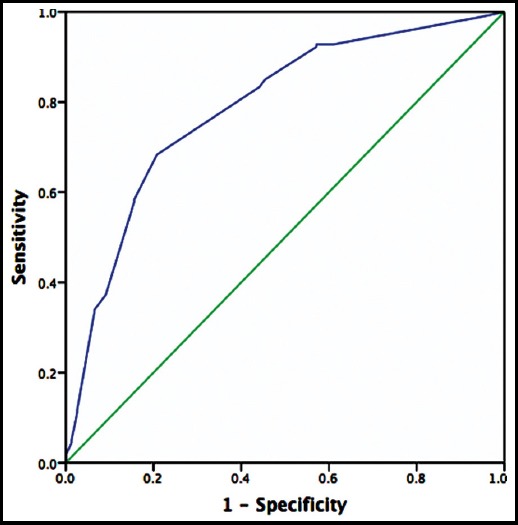
ROC curve showing the ability of MUAC to diagnose children with moderate and severe acute malnutrition in children aged 6-59 months (AUC= 0.786 [95%CI; 0.725-0.848]).

A total of 188 (73.2%) children were identified as SAM by MUAC as compared to WHZ-score 180 (70%) (Table-I). Both MUAC and WHZ-scoring showed significant association to diagnose MAM and SAM among children aged 6–59 months (p-<0.001). In Fig.1 MUAC curve illustrate the sensitivity and specificity of MUAC for diagnosis of SAM according to WHZ <-3SD. ROC curve analysis for MUAC (cut-off value 103, 95%CI; AUC: 101-107 mm) showed it as an excellent predictor (p=<0.001) for child having SAM, with area under curve (AUC= 0.786 [95%CI; 0.725-0.848]).

## DISCUSSION

Under-nutrition in children requires instant nutrition rehabilitation and medical attention as it can be a lethal condition if not timely diagnosed. Therefore, it is very important to identify these fragile and vulnerable children at its earliest in order to provide them nutritional support. Malnutrition mainly effects younger children. In our study the mean±SD age for the study participants was 13.43±8.81 months, comparable with a study published in PLoS One in which a median age of 15 months (IQR: 10–22 months).^7^ A higher mean and median age was documented in various studies showing 26.36±13.9 months and 23 (IQR 12–37).^2^,^4^ Majority of our children were in the age group of 6-11 months (n=130; 50.5%) followed by 12-23 months (n=96; 37.4%). A study from India by Sachdeva showed 24.5% were in the age range of 6–11 months but a study published in Nutrient depicted more children in the age range of 36-59 months (50%).^3^,^4^

Maternal education has an important and crucial role related to feeding practices, child malnutrition and child survival. In our study (n-137; 53.3%) mothers were illiterate. Similarly, a study on malnourished children published in Nutrient showed maternal illiteracy in 63% of mothers.^3^ In a research from Ghana it was documented that with increase in mother’s years of education and body mass index there is decreased risk of malnutrition in under 5 children.^8^ Several studies revealed that SAM was associated with maternal illiteracy and sub-optimal frequency of complementary feeding.^9^,^10^

According to the World Health Organization a major factor for almost one-third of the cases of malnutrition is inappropriate feeding in children. In our study, exclusive breast feeding rate is quite low (n-82; 32%). Results of a study from china documented that for children < 36 months shorter duration of breastfeeding and low rates of exclusive breastfeeding among children < 6 months of age is a major reason for malnutrition and stunting.^11^

WHZ and MUAC are used to assess nutritional status of children. Although both anthropometric indicators are used to assess the same problem but the use of only one method may lead to misdiagnosis of few cases. Contradicting it our analysis revealed that almost similar percentage of children were categorized as SAM by WHZ or MUAC criteria (70% vs 73.2%) respectively. Our results are comparable with a study published by Chiabi et al, showing AUC was greater for MUAC [0.809 (95% CI, 0.709-0.911, p = 0.001)] than WHZ [0.649 (95% CI, 0.524-0.774, p = 0.032)]. Moreover, MUAC is a better predictor of death than WHZ.^12^ A study from India documented that measurement of MUAC was found to correspond with WHZ in mild to moderate malnutrition (87.5%) but in severe malnutrition it was low (70%).^13^ When single MUAC cut-off (< 12.5 cm), and WHZ < -2SD were compared in a study done by Mogendi there was no statistically significant difference in sensitivity and specificity.^14^ A study from Kenya showed that for predicting death, area under the ROC curves did not differ significantly between two indicators (MUAC: 0.75 [95% confidence interval, 0.72-0.78]; WHZ: 0.74 [95% confidence interval, 0.71- 0.77]) (*P* = 0.39).^15^

Although the level of wasting was low in a study by Dukhi N who compared these two methods for evaluating the nutritional status of under 5 children the results revealed that more children (44) were identified to be malnourished based on WHZ score as compared to MUAC (38). This study showed WHZ method as a more sensitive measure of child malnutrition as more than double children identified to having SAM compared to MUAC.^16^

Extensive literature searching revealed several reports showing a discrepancy between children who fall below the cut-off points of MAM and SAM using WHZ or MUAC criteria. A study published in BMC from 47 different countries showed that in the developing world a great variation exists for diagnosis of malnutrition in children based on WHZ score or MUAC. In some countries the substantial number of children are diagnosed by using MUAC and in others WHZ score criteria.^17^ Conflicting with our results a study from Ethiopia showed that more children are categorized as wasted by MUAC (10.5%), as compared with WHZ (5.4%).^3^

## CONCLUSION

Both MUAC and WHZ-scoring showed fair degree of agreement to diagnose moderate and SAM among children aged 6-59 months. This may have important implications for community diagnosis and management of moderate to severe acute malnutrition. MUAC can be used as a rapid diagnostic tool for quick assessment and referral of malnourished children for nutritional rehabilitation.
